# Annotations

**Published:** 1904-08-27

**Authors:** 


### ANNOTATIONS.

### The Premier of New Zealand and Child-life Preservation.

Whatever differences of opinion there may be as to some of the views of the Premier of New Zealand, his vigour and earnestness are beyond question. He is, in a true sense, an alive man, and any proposals he makes in the interests of the colony in which he holds the foremost official position, therefore, merit consideration. The state of affairs disclosed in the report of the New South Wales Royal Commission on the falling-off of the natural increase of children prompted Mr. Seddon to have prepared a return on the subject for New Zealand. This return, which has satisfied him that a very large number of the 20,000 infants whose lives were lost in a period of 10 years might have been saved, has convinced the Premier and his colleagues that an opportunity should be given to the New Zealand Parliament of dealing with the matter; and Mr. Seddon, in a memorandum he has issued, advocates characteristically courageous measures. In the first place he urges that a Bill for the compulsory registration of midwives should be passed, with a provision for the supply in each centre of midwives to attend the wives of the poor gratis. He then proceeds to suggest the establishment, by the State, of maternity homes and foundling hospitals, the payment by the State of nurses for the sick poor in their homes, and also of the fees for the training of such nurses; the erection by the State of day homes for infants; a drastic law to prevent the insurance of children of tender years for more than £5; and the repeal of the law allowing persons responsible for the maintenance of illegitimate children to commute the weekly or monthly amount necessary for the purpose. In recommending these schemes Mr. Seddon admits that in older countries the benevolent and philanthropic might render some of them superfluous or undesirable, but in New Zealand, he contends that, there being no leisured classes and few people of ample means, the responsibility of saving the lives of the children properly devolves upon the State. The reception of them in the colony will be awaited with interest here. We do not know how far it is true that too much money is spent in New Zealand "in coffins, head-stones, and funeral expenses," but there is force in the final remark of the Premier, that "to bewail the want of a proper natural increase is pure hypocrisy unless something substantial is done in the way of saving the infant life that is born."

### Complexion and Disease.

The old doctrine of the temperaments in relation to disease has no considerable influence on medical teaching and theory at the present day. Its vigour has to a large extent declined, perhaps not with altogether good results, under the strenuous competition of the more precise methods of the clinical laboratory and the exact discipline of physical examination. In one of the discussions at the recent meeting of the British Association, however, one of the speakers made a contribution which certainly recalls some of the articles of the older faith in reference to the relationship between disease and the inherited personal qualities of individuals. It was shown that persons of the blonde type suffer more than do those of the brunette type from rheumatic diseases such as tonsillitis, acute rheumatism, and heart disease, while the reverse is the case in reference to pulmonary tuberculosis, nervous disorders (particularly epilepsy), and cancer. Further, this influence has an age-relationship, and disease during early life is found to fall more heavily on children of blonde complexion. Thus in overcrowded areas the proportion of the brunette element is in excess, and the infant mortality amongst blonde children is greatest. This is in some measure corrected by the great mortality at ages from 20 to 25 due to tuberculosis—a disease which, as already stated, falls in undue proportion on those of dark complexion. These tendencies appear to be accentuated by town residence; hence the conditions of modern life would seem to favour a dark-haired population and to be antagonistic to a fair-haired population. What effect this is likely to have on the race-characters of the nation the future alone can disclose; but if the prophets are accurate, it is inevitable that sooner or later we shall be unable to claim the compliment—*Non Angli, sed Angeli.*

### Fatal Poisonings in 1902.

Amongst the varied statistical information provided by the Registrar-General of Births, Deaths, and Marriages in England and Wales in his official report for 1902 may be found a tabulated statement showing the number and causes of the deaths from poisoning which occurred during that year. The number of deaths certified under this heading was 1,195, being a decrease of 2 as compared with 1901. These include 150 deaths due to anæsthetics administered for operation purposes. Of these the great majority—namely 101—were due to chloroform, 9 to ether, 5 to a mixture of chloroform and ether, 6 to the A.C.E. mixture, 1 to nitrous oxide, 5 to that of gas and ether combined, and in 22 the form of anæsthetic was not specified; among this group, rather curiously, is placed 1 death due to atropine. Suicidal poisonings proved fatal in 503 instances, and of these no fewer than 140 were attributed to carbolic acid, the deaths in the previous year from the same cause being 142. The other most attractive poisons to would-be suicides proved to be oxalic acid with 64 deaths, opium and other narcotics with 56 deaths, hydrochloric acid with 43 deaths, and prussic acid and oil of bitter almonds with 31 deaths. Regarding the sex-relationship of the 503 fatal suicidal poisonings, 294 occurred in males, and 209 in females. There are some curious disparities to be noted when the sex predilection for individual poisons is studied. Thus, carbolic acid was used by 77 females and 63 males; ammonia shows 7 females to 3 males; and in strychnine the numbers are 7 females, 4 males. On the other hand, the males show some excess in oxalic acid (37 to 27), and hydrochloric acid (29 to 14), whilst in sulphuric acid the numbers are equal (3 of each). The disproportion is decided in opium poisoning (38 males, 18 females), and is still more marked in the case of prussic acid (29 to 2) and cyanide of potassium (18 to 1). Of 6 deaths due to chlorodyne, all occurred in males. The deaths attributed to murder by poison were 2 in number; in one antimony was used, in the other carbolic acid.

